# Current Status and Future Potential of Energy Derived from Chinese Agricultural Land: A Review

**DOI:** 10.1155/2015/824965

**Published:** 2015-03-22

**Authors:** Ningning Zhai, Chunlan Mao, Yongzhong Feng, Tong Zhang, Zhenjie Xing, Yanhong Wang, Shuzhen Zou, Dongxue Yin, Xinhui Han, Guangxin Ren, Gaihe Yang

**Affiliations:** ^1^College of Agronomy, Northwest A&F University, P.O. Box 95, Yangling, Shaanxi 712100, China; ^2^The Research Center of Recycle Agricultural Engineering and Technology of Shaanxi Province, Yangling, Shaanxi 712100, China; ^3^College of Forestry, Northwest A&F University, Yangling, Shaanxi 712100, China

## Abstract

Energy crisis is receiving attention with regard to the global economy and environmental sustainable development. Developing new energy resources to optimize the energy supply structure has become an important measure to prevent energy shortage as well as achieving energy conservation and emission reduction in China. This study proposed the concept of energy agriculture and constructed an energy agricultural technical support system based on the analysis of energy supply and demand and China's foreign dependence on energy resources, combined with the function of agriculture in the energy field. Manufacturing technology equipment and agricultural and forestry energy, including crop or forestry plants and animal feces, were used in the system. The current status and future potential of China's marginal land resources, energy crop germplasm resources, and agricultural and forestry waste energy-oriented resources were analyzed. Developing the function of traditional agriculture in food production may promote China's social, economic, and environmental sustainable development and achieve energy saving and emission reduction.

## 1. Introduction

With the rapid growth of the global economy, the world's energy demand will increase from approximately 12 billion tons of oil equivalents (t.o.e.) in 2009 to 17 billion or 18 billion t.o.e. by 2035. China is the second largest economy worldwide and has surpassed the US as the world's largest energy-consuming country; the energy consumption of China has reached 2.61 billion t.o.e. [1]. Consequently, the role of demand and supply of traditional fossil energy resources has become increasingly important in international relations [2, 3]. Meanwhile, carbon dioxide emissions are expected to increase from 29 Gt/yr to 36 Gt/yr or 43 Gt/yr [4], which will put great pressure on fossil energy resources and significantly affect global climate [5]. China is the largest emitter of greenhouse gases worldwide and contributed more than half of the global carbon dioxide emissions from 1990 to 2008 [6, 7]. With the establishment of the energy conservation and emission reduction target of the international community, the contradiction between economic growth and energy consumption in China has become increasingly serious [8]. Thus, energy consumption on global climate change and human survival has become increasingly critical, and energy shortage will be an important restrictive factor in economic development [9]. At present, these problems are the biggest threat to humanity.

Numerous reports on global climate change have been published [2, 10], including the Kyoto Protocol [11]. Many intergovernmental actions have already been implemented to solve these problems, such as the Intergovernmental Panel on Climate Change. These intergovernmental actions on energy sources include wind, hydro-, and biomass [12]. The Chinese government has also launched an energy project to develop renewable energy sources, such as solar, wind, hydro-, and biomass, to optimize the existing energy structure to prevent energy shortage [13].

In recent years, China has achieved considerable progress in utilizing and developing new energy resources. Based on the 12th Five-Year Plan of China, nonfossil fuel should account for 11.4% and 20% of the total primary energy consumption by 2015 and 2020, respectively. At the end of 2011, the installed generation capacity of nonfossil energy of China was 27.5%, which comprised hydropower (21.7%), nuclear power (1.19%), wind power (4.27%), and biomass power (0.41%) [14]. The development of biomass energy is relatively slow in China, but the new energy field has great potential because of its minimal negative effect on the environment, low cost of raw material, wide distribution of resources, and great energy conversion potential [14, 15]. Agricultural biomass is a preferred alternative energy source to overcome these challenges because it is environment friendly and accessible [1, 16, 17].

Agriculture is an old industry and the main provider of energy in rural areas of developing countries. In addition to providing food, clothing resources, and household energy, agriculture also provides various sources of raw materials to produce energy in the form of energy agriculture, such as oil, starch, sugar, straw, algae, trees, and livestock feces [18]. Through modern processing technologies, biomass energy production can be converted to fuel oil, gas, or electric energy, which can optimize the energy supply structure, reduce greenhouse gas emissions, and maintain urgent economic and social sustainable development. In this study, energy agriculture is an energy model to reduce CO_2_ emissions and adjust the energy demand structure [11].

Energy agriculture has started earlier and developed more rapidly in other countries than in China. Since 1975, Brazil has implemented a plan that massively produces fuel alcohol using sugar cane [19, 20]. Recently, developed countries, such as the US, Japan, Canada, UK, and Germany, as well as developing countries, such as the Philippines, Indonesia, and Thailand, have formulated their own biofuel development programs [21]. From 2000 to 2005, ethanol production worldwide increased from 4.6 billion gallons to 12.2 billion gallons, a jump of 165% [22]. The biofuel production of Europe increased from 80 thousand tons in 1992 to 470 thousand tons in 1998 and that of the US rapidly increased from 30.28 million tons in 1979 to 3.63 billion tons in 1990, 4.54 billion tons in 1999, and 9.69 billion tons in 2003. The study on biodiesel of the US began in 1983, and its biodiesel production reached 450 million gallons in 2007, which increased by 80% compared with the production in 2006 [23]. The US also planned to increase the annual output to 6.1 million tons by 2015. Other countries are also actively and rapidly promoting the development of the industry, including Germany, Japan, Brazil, and India [24].

Bioenergy in China was not studied as early as the other countries. Energy agriculture had not been given close attention by law until 2006 with the establishment of the “renewable energy law” [13]. Excess rice and agricultural waste are the main raw materials of biomass energy, but the cultivation area of energy crops is continuously expanding [25]. Similarly, investigation on biodiesel produced by waste cooking oil, oil extraction residue, and forest oil fruit also started late in China [24]. Nevertheless, China has started emphasizing on energy agriculture and biomass energy industry and developed a clear program [26].

In the National Long-Term Development Plan (2005–2020), the “agricultural and forestry biomass project” is classified as a major project and an important part of the national energy strategy. The State Forestry Administration has conducted a preliminary plan for biomass energy aimed at developing 13.33 million hectares of forestland to grow biomass energy raw materials. During the 11th Five-Year (2006–2010) period, China had cultivated biomass energy plants to meet the supply requirement of 6 million tons of raw diesel and raw materials for 15 million KW installed capacity of power generation [27]. In April 2007, the Department of Science and Technology Education of the Ministry of Agriculture released an investigation and assessment letter to all provinces and cities regarding the development of suitable energy crop resources in marginal land. The investigation and assessment work started with winter fallow land that could be used for planting energy crops. The implementation of this measure will promote the planning and development of energy agriculture [28].

The development of agricultural energy in China is promising. China is currently one of the world's largest biogas production countries [29] and has set up a standard system of biomass solid fuel. About 19 agriculture industry standards presently exist in China. By the end of 2010, the number of promulgated and implemented energy standards in Chinese rural areas reached 94 [30]. At the beginning of the 21st century, China established several production enterprises and set relevant standards to develop fuel ethanol; thus, fuel ethanol has been used in cars in some provinces for several years [27]. Energy agriculture has not attracted attention in China because the policies focus on reusing excess rice and agricultural waste [31]. In the next decade, China will be focusing more attention on the energy agriculture industry, including its scientific input and policy orientation.

## 2. Energy Supply and Demand of the Chinese Energy Agriculture Development

### 2.1. Structure Characteristics of the Energy Supply and Demand

Table 1 shows the trend of the energy supply and demand of China from 1978 to 2012. According to statistics, raw coal output increased from 70.3% in 1978 to 76.5% in 2012 and showed a dominant part in China's energy supply structure despite the occurrence of certain amplitude fluctuations during this period. Meanwhile, the self-supply capacity of oil decreased from 23.7% in 1978 to 8.9% in 2012, whereas natural gas and electricity increased from 2.9% to 4.3% and 3.1% to 10.3%, respectively, in more than 30 years from 1978.

Coal consumption accounted for 76.2% of the total energy consumption in 1990, followed by a decrease of 9.6% to 66.6% in 2012. In the same period, production only was 76.5% (2012), which indicates that supply exceeded demand. The proportion of crude oil consumption decreased from 22.7% to 18.8% in 2011, which indicates a decrease of 3.9%. This result presents a great contradiction of supply and demand. The output production of natural gas increased from 1.9% in 1994 to 4.3% in 2012, and the consumption proportion increased by 1.8% in 1997 to 5.2% in 2012. The output of rapidly developing wind power, hydropower, and nuclear power increased from 3.1% in 1978 to 10.3% in 2012, which was equivalent to 3.3 times that in 1978, and consumption was equivalent to 2.8 times that in 1978, indicating an oversupply problem [31]. However, regional and seasonal power supply and demand contradiction still exists. Therefore, optimizing China's energy structure will become a priority in future development.

### 2.2. China's Dependence on International Energy Market

**Figure 1:**
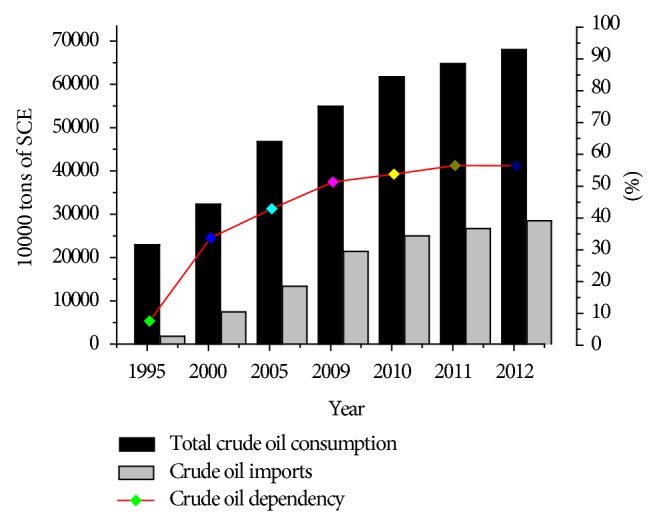
Consumption and imports of crude oil in China [32, 45].

After more than half a century of exploration and development, China's energy industry has achieved significant success. However, China still shows an unbalanced relationship of energy supply and demand (Table 1) and relies on foreign imports, particularly oil. China's dependence on crude oil imports from the international crude oil market was only 7.59% in 1995, but the proportion increased continuously to 33.76% in 2000, 42.9% in 2005, and 51.3% in 2009. The foreign dependence indicated an increase at an average rate of 3% annually. In 2010, China's dependence on foreign oil remained high, but the increase was only 2.5%, which was lower than the average rate 15 years ago. The latest data shows that the crude oil dependency proportion has reached 56.5 in 2011 and 2012 (see Figure 1). The International Energy Agency forecasted that China's dependence on oil imports will reach 76.9% in 2020 [31, 32]. Based on these findings, China's energy supply will continue to rely on the international crude oil market for a long time, and the dependence rate will further increase. Therefore, changing and optimizing the energy structure at the national strategic level are significant.

## 3. Framework of China's Energy Agricultural Development Strategy

### 3.1. Basic Concept, Definition, and Characteristics of Energy Agriculture

**Figure 2:**
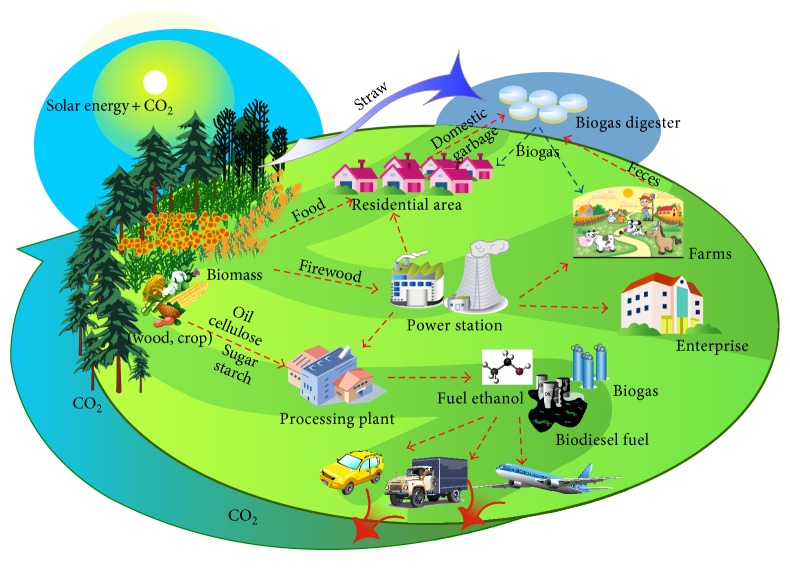
Diagram of energy agriculture concepts and characteristics.

Cultivating plant oil sources is the best explanation for energy agriculture, which is an academic concept that appeared in recent years. The first Chinese official publications on energy agriculture were “Shallow Theory of the Development of China's Energy Agriculture” by Yao [33] and “Necessity and Strategy of Development of Energy-Oriented-Agriculture” by Feng et al. [34]. Both studies discuss the concept of energy agriculture. Yao regarded energy agriculture as agricultural production and related activities for providing energy resources and transforming products. Feng et al. reported that energy agriculture aims to convert solar energy into an energy that can be used directly. The energy agriculture proposed by Lu aims to produce energy [35]. Hu and Gu [36] considered that energy agriculture includes various energy utilization patterns; the main development resource was biomass energy. According to Li [37], energy agriculture is a motivation to produce high-value crops with high biomass energy content. Biomass energy locked in biomass crops will then be translated into energy that can be directly used for economic and social development. Xu et al. and Sun et al. [21, 38] generally regarded that energy agriculture is the foundation of biomass industry and a new form of agriculture that supplies raw materials to produce biomass energy. Based on previous studies and the special role of agriculture in solving energy problem, energy agriculture can be defined as an agricultural production activity, where solar energy is fixed through photosynthesis by green energy crops and then converted into energy that can be directly used by humans (Figure 2). Energy agriculture has the following characteristics.

1. Energy agriculture is a concept that involves interdisciplinary industry of planting, breeding, and modern energy chemical industry, as well as equipment manufacturing, electric power, and transportation. Energy agriculture also produces living resources, such as food, clothing, and housing, as well as energy. It is an integrated science based on land, crops, and artificial regulation. This concept belongs to agriculture category because the process and utilization of crops depend on land, similar to traditional planting and breeding.
2. Energy agriculture mainly solves the contradiction between fossil energy consumption and development of the environment and economy in the human development process. The energy provided by energy agriculture is different from the traditional primary energy, such as coal, oil, and natural gas. This energy is renewable, which is fixed through photosynthesis of green plants and stored as organic energy in the body of crops.
3. Energy agricultural production cannot be used directly and must be combined with certain processing technologies. The energy can only be used by humans after being converted under certain technical and economic conditions rather than through direct combustion of straws or firewood.
4. Production activities of energy agriculture are very systematic. They include a series of production and research activities, such as energy crop cultivation and management, energy crop germplasm resource selection and breeding, processing technologies of energy agricultural production, equipment design, and development.
5. Fixing atmospheric CO_2_ is the main production process of energy agriculture. CO_2_ and H_2_O are released from the process of exploiting agriculture products. CO_2_ emissions do not increase because of energy flow and material recycling in the ecosystem. Therefore, developing energy agriculture is suitable for controlling the concentration of greenhouse gases in the atmosphere. This process is also significant for controlling the increase of surface temperature and maintaining the ecosystem carbon balance of the earth.
6. The prerequisite for energy agriculture development is food and energy security, which are the major challenges faced by energy agricultural development. Energy agriculture can be performed smoothly by guaranteeing food security.

### 3.2. Technology System for Energy Agriculture in China

**Figure 3:**
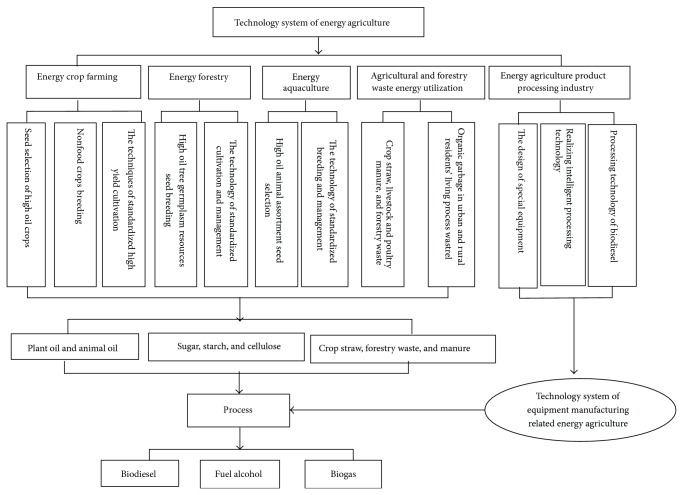
Technology system of energy-oriented agriculture.

According to its definition and characteristics, energy agriculture involves five sectors, namely, energy crop farming, energy forestry, energy animal husbandry, utilization of waste produced in agricultural production, and related processing industry (Figure 3) [39]. The contents of each sector are as follows.

The technology system of energy crop is nonfood crops breeding and high-yield culture technique. Nonfood crops include starchy crops, oil crops, and sugar crops. Energy forestry is cultivation and breeding technology system of germplasm resources, including firewood forest-based wood fuel, woody oil, and woody starch crops. Woody oil crops include* Jatropha*, tung tree,* Pistacia*,* Xanthoceras sorbifolia* Bunge, and oil tea camellia. Woody starch crops are mainly oak fruit (acorn).

Energy technology system in animal husbandry uses modern biobreeding techniques to directly breed existing animals with high grease conversion rate, such as pigs. This technology aims to cultivate new species of animals that can efficiently convert plant products, such as straw, wheat bran, and other nonfood products, into axunge for biodiesel processing [40]. Currently, herbivores are preferred, such as pigs and sheep.

Energy utilization technology system is a technological system for producing biogas, biodiesel, and cellulosic fuel ethanol from wastes, such as various straws, tree branches, feces, and kitchen waste, produced by farming, animal husbandry, or humans.

The manufacturing technology system of energy agricultural equipment involves designing special equipment and exploring intelligent processing technologies. Equipment and technology are mainly used to utilize energy agriculture, forestry, animal husbandry wastes, and so on. The equipment and products of producing biodiesel oil, and fuel ethanol are all included in the system (Figure 3).

### 3.3. Development Potential of Chinese Energy Agriculture

According to the policy and tendency of China's energy development, the priorities of energy development in China are the utilization of marginal land resources, selection and cultivation of energy biomass germplasm resources, and efficient utilization of waste energy in the production process.

#### 3.3.1. Potential of Marginal Land Resources

China has a vast territory. In addition to the 121 million hm^2^ of cultivated land used to ensure the nation's grain production, China's available nonarable land area is large. According to the survey, China has 108 million hm^2^ of uncultivated land, and 35.35 million hm^2^ is suitable for agriculture, accounting for 32.7% of the total wasteland area. The total wasteland area may be equivalent to 36.9% of the existing arable land area. Forestry land covers 267.43 million hm^2^, but only 76.62 million hm^2^ of waste mountains and land is suitable for tree planting, accounting for 28.6% of the woodland area. The woodland area is equivalent to 6.2% of the existing forest area [28].

According to the information from the Science and Research Department of the Ministry of Agriculture (Science and Education Division of the Ministry of Agriculture) in April 2007, 34.2 million hm^2^ of noncultivated land suitable for cultivation of energy crops is distributed in 1845 counties (cities and regions) of China. Approximately 26.8 million hm^2^ of wasteland is available for farming. First-, second-, and third-class wastelands cover 4.33 million hm^2^ (16.2%), 8.73 million hm^2^ (32.6%), and 13.73 million hm^2^ (51.2%), respectively [41]. The amount of winter-free farmland is approximately 7.4 million hm^2^.

Considering crop ecological adaptability, the suitable wasteland areas for planting sweet sorghum, cassava and sugar cane are approximately 13, 5, and 15 million hm^2^, respectively. If 20% to 30% of wastelands are cultivated with energy crops, the biomass energy production can be converted to 50 million tons of alcohol based on the existing technologies in China [41].

#### 3.3.2. Potential of Germplasm Resources

According to the notice of “strengthening biological fuel ethanol project construction and management” jointly enacted by the National Development and Reform Commission and Ministry of Finance in 2006, as well as “the guidance of promotion of the healthy development of deep processing of corn” and “development planning of agricultural biomass energy (from 2007 to 2015)” promulgated by the Ministry of Finance China and the Department of Agriculture in 2007, the development of energy crops in China must adhere to the precondition of “do not compete for food with people, do not compete for land with food” [42] Therefore, the energy agricultural germplasm resources of China aim to develop nonfood energy crops. Approximately 40 kinds plants can be used as energy crops, including short rotation trees, herbaceous crops, sugar crops, vegetable oil crops, and plants used for extracting hydrocarbons, such as sugar beets, sugar cane, sweet sorghum,* Miscanthus* crop, sweet corn, beans, peanuts, cotton, sunflower, rapeseed, palm, and castor. Studies show that the highest per unit yield is achieved by sugar cane and sweet sorghum. The biomass per unit area produced by sugar cane and sweet sorghum is 12 times as much as maize and 2.5 times as much as sweet potato and cassava. Maize is the most suitable crop for fuel ethanol production because 2.82 tons of maize can produce 1 ton of fuel ethanol. However, the fuel ethanol production per unit of land of maize is the lowest, whereas sugarcane is the highest, followed by sweet sorghum, cassava, and sweet potato. Maize is suitable for planting in almost all parts of China, cassava and sugarcane are mostly planted in southern China, and sweet potato and sweet sorghum are suitable in southwestern and northern China [43].

According to the results of the sixth forest resource inventory published in 2005, the national forest area is 175 million hm^2^, with total standing volume of 13.62 billion m^3^ and total woody biomass resources of more than 18 billion tons. Energy forestry mainly contains woody biomass fuel resources, woody oil plant resources, woody starch, and fiber plant resources.

China's existing firewood forest area is approximately 3.03 million hm^2^, which can provide 21.24 million tons of firewood that can replace 12.11 million tons of coal equivalents. Preliminary estimates showed that approximately 60 million hm^2^ of shrubs can be planted in western China. If 60% of these areas are used as energy forest, approximately 144 million tons of biomass can be obtained (if 4-ton biomass is produced per hectare annually), which can replace 93.6 million tons of coal equivalents.

Approximately 3.43 million hm^2^ of woody oil crop area has been planted in China. A total of 151 energy oil plants (seed plant), 697 genera, and 1554 species exist, which account for 5% of the total seed plants in China. About 154 species of woody plants have seed oil content of more than 40%, and up to 10 species are suitable for building raw material bases and scale supply bases for improved variety using barren hills and sand.  Table 2 shows the main woody oil crops.

Some inedible woody fruit trees are rich in starch; for example, the acorn of oak comprises 50% starch. The existing area of oaks is up to 18 million hm^2^ in China, and more than 6.7 million hm^2^ of oak area exists in Inner Mongolia, Jilin, and Heilongjiang provinces. More than 10 million tons of fruits can be harvested from these oaks annually, which contain more than 5 million tons of starch. If these fruits are fully utilized, 2.26 million tons of biological fuel alcohol can be produced. Hemp and other lignocellulose materials are widely distributed in China. The suitable planting areas are north to Heilongjiang, south to Hainan, west to Xinjiang, and east to coastal areas, which cover up to 1.06 million hm^2^. The dry matter is up to 23 tons per hectare annually, and the lignocellulose content is as high as 68% to 75%. If each ton of lignocellulose can produce 0.35 tons of industrial alcohol, hemp (dry matter basis) per hectare can produce 5.75 tons of industrial alcohol average annually.

#### 3.3.3. Potential of Agricultural Waste Resource

According to the information provided by the China Statistical Yearbook in 2012, the estimated amount of crop straw in China was 972.26 million tons in 2011. Food crop straw, oil crop straw, and other crop straws were 765.32, 60.15, and 146.79 million tons, respectively. The dung discharge was about 39.87 billion tons, in which about 54.5% was pig manure (21.7 billion tons) and 38.69% was cow dung (15.4 billion tons). Human feces emission in 2011 was about 25.73 million tons [44].

**Figure 4:**
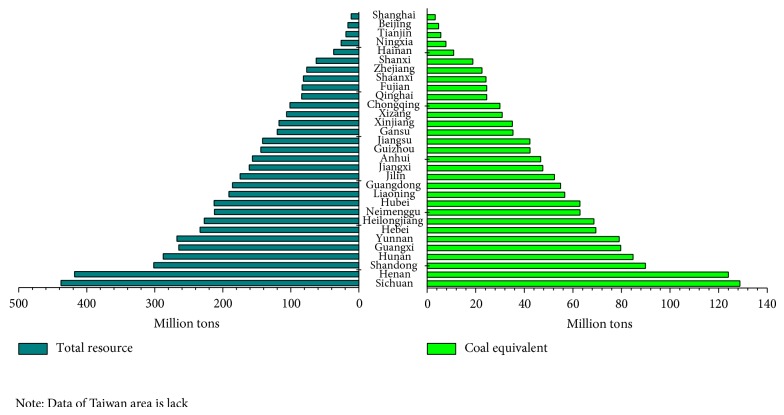
Resource and potential of agricultural waste of province's level in mainland China [20, 31].

Among all the provinces and cities in China, Guangxi, Heilongjiang, and Henan were the top producers of crop straw. Xizang, followed by Shanghai and Qinghai, produced the lowest quantity of crop straw. Sichuan ranked first in terms of livestock manure emission, followed by Henan and Hunan. Shanghai, behind Beijing and Tianjin, was the lowest in the rank. Given these data, Sichuan ranked first in agricultural waste potential, and its total quantity of agricultural waste was 437.88 million tons, accounting for 8.8% of the whole country, followed by Henan and Shandong, accounting for 8.4% and 6.1%, respectively. Shanghai ranked last in resource potential because its quantity of agricultural waste was 11.26 million tons, accounting for 0.23%, followed by Beijing and Tianjin with 0.32% and 0.38%, respectively (Figure 4) [31, 44].

When these straws and dung were used for digestion at 35°C, the biogas yield potential of crop straws in 2011 was estimated to be 311.97 billion m^3^, which is equivalent to 220 million tons of standard coal. The potential of livestock manure was 288.93 billion m^3^, which is equivalent to 205 million tons of SCE (standard coal equivalent). The biogas potential of human feces was 12.5 billion m^3^, which is equivalent to 8.8 million tons of SCE. The total biogas production potential of agricultural waste in 2011 was 613.43 billion m^3^ or 436 million tons of SCE, accounting for 17.6% of the coal produced in the same period.

The above analysis showed that China has considerable reserves of energy agriculture resource that can potentially produce large amounts of energy when used well.

## 4. Conclusions

1. China's energy agricultural development and energy supply and demand structure from 1978 to 2012 showed an increasing demand for renewable energy and decreasing demand for raw coal and oil. The supply-demand contradiction of raw coal and oil still continued, and an oversupply of the overall performance of gas and electricity existed.
2. China's dependence on foreign oil continued to increase for a long time. Thus, strategies on optimizing and changing the energy structure must be planned at the national level.
3. Energy agriculture is a special agricultural production activity, where solar energy is fixed through photosynthesis by energy crops and then converted into energy that can be directly used. An energy agriculture technology system was also established based on energy crop, energy forestry, energy animal husbandry, energy utilization of agricultural waste, and equipment manufacturing.
4. China has an immense energy potential for marginal land resources, germplasm resources, and agricultural and forestry wastes. Developing energy agriculture has no limits if resources are fully utilized. Meanwhile, the ecological construction of China will improve with combined energy agriculture.

## Figures and Tables

**Table 1:** Production and consumption of China's energy structure [45].

Year	Total energy production (10000 tons of SCE)	As percentage of total energy Production				Total energy consumption (10000 tons of SCE)	As percentage of total energy consumption			
		Coal	Crude oil	Natural Gas	Hydropower, nuclear power, wind power		Coal	Petroleum	Natural gas	Hydropower, nuclear power, wind power
1978	62770	70.3	23.7	2.9	3.1	57144	70.7	22.7	3.2	3.4
1980	63735	69.4	23.8	3	3.8	60275	72.2	20.7	3.1	4
1985	85546	72.8	20.9	2	4.3	76682	75.8	17.1	2.2	4.9
1990	103922	74.2	19	2	4.8	98703	76.2	16.6	2.1	5.1
1991	104844	74.1	19.2	2	4.7	103783	76.1	17.1	2	4.8
1992	107256	74.3	18.9	2	4.8	109170	75.7	17.5	1.9	4.9
1993	111059	74	18.7	2	5.3	115993	74.7	18.2	1.9	5.2
1994	118729	74.6	17.6	1.9	5.9	122737	75	17.4	1.9	5.7
1995	129034	75.3	16.6	1.9	6.2	131176	74.6	17.5	1.8	6.1
1996	133032	75	16.9	2	6.1	135192	73.5	18.7	1.8	6
1997	133460	74.3	17.2	2.1	6.5	135909	71.4	20.4	1.8	6.4
1998	129834	73.3	17.7	2.2	6.8	136184	70.9	20.8	1.8	6.5
1999	131935	73.9	17.3	2.5	6.3	140569	70.6	21.5	2	5.9
2000	135048	73.2	17.2	2.7	6.9	145531	69.2	22.2	2.2	6.4
2001	143875	73	16.3	2.8	7.9	150406	68.3	21.8	2.4	7.5
2002	150656	73.5	15.8	2.9	7.8	159431	68	22.3	2.4	7.3
2003	171906	76.2	14.1	2.7	7	183792	69.8	21.2	2.5	6.5
2004	196648	77.1	12.8	2.8	7.3	213456	69.5	21.3	2.5	6.7
2005	216219	77.6	12	3	7.4	235997	70.8	19.8	2.6	6.8
2006	232167	77.8	11.3	3.4	7.5	258676	71.1	19.3	2.9	6.7
2007	247279	77.7	10.8	3.7	7.8	280508	71.1	18.8	3.3	6.8
2008	260552	76.8	10.5	4.09	8.62	291448	70.3	18.3	3.7	7.7
2009	274619	77.3	9.9	4.1	8.7	306647	70.4	17.9	3.9	7.8
2010	296916	76.5	9.8	4.2	9.4	324939	68.0	19.0	4.4	8.6
2011	317987	77.8	9.1	4.3	8.8	348002	68.4	18.6	5.0	8.0
2012	331848	76.5	8.9	4.3	10.3	361732	66.6	18.8	5.2	9.4

Note: The coefficient for conversion of electric power into SCE is calculated on the basis of the data on the average coal consumption in generating electric power in the same year.

**Table 2:** Biological characteristics of mainly woody fuel.

Tree name	Harvest organ	Yield kg/hm^2^	Oil content	C16-C18 FA	Distribution area
*Jatropha curcas* L	Seed	9750	More than 39.8% (kernel 64.5%)	100.00%	Guangdong, Guangxi, Yunnan, Guizhou, and Sichuan provinces etc.
*Xanthoceras sorbifolia* Bunge	Seed	9000	More than 35.5% (kernel 66.4%)	88.97%	North China, East China, and Northwest China
*Pistacia chinensis* Bunge	Seed	12000	35.1% (kernel 56.5%)	100.00%	North to Yellow River basin, south to Guangdong, Guangxi
*Cyperus esculentus* L	Tuber	6000	25.30%	100.00%	North to inner Mongolia, south to Jiangsu and Zhejiang provinces
*Euphorbia lathyris* L	seed	1650	More than 43.5% (kernel 69.2%)	99.70%	North to Jilin, south to Jiangsu and Zhejiang provinces
*Swida wilsoniana *	seed	12000	33%~36% (nutlet 55%–59%)	77.68%	Yellow River basin regions to their south, Hunan, Jiangxi, and Hubei provinces are the main areas
